# Overcoming Barriers in the Surgical Management of Osteogenesis Imperfecta: Insights From a 13-Year Retrospective Cohort

**DOI:** 10.7759/cureus.85610

**Published:** 2025-06-09

**Authors:** Hamza Bensaghir, Sarah Hosni, Chaimae Ben Driss, Loubna Aqqaoui, Houda Oubejja, Sidi Zouhair Fellous El Alami, Fouad Ettayebi, Tarik El Madhi

**Affiliations:** 1 Pediatric Surgical Emergency Department, Children Hospital of Rabat, Faculty of Medicine and Pharmacy, University Mohamed V, Rabat, MAR; 2 Pediatric Orthopedic Surgery Department, Children Hospital of Rabat, Faculty of Medicine and Pharmacy, University Mohamed V, Rabat, MAR

**Keywords:** intramedullary rodding, osteogenesis imperfecta, pediatric orthopedics, skeletal dysplasia, telescopic nailing

## Abstract

Osteogenesis imperfecta (OI) is a rare genetic disorder characterized by bone fragility and reduced bone mass. This retrospective study analyzed 42 cases of OI treated at the pediatric traumatology and orthopedics department over a 13-year period. The study aimed to examine the clinical and radiological aspects associated with osteosynthesis, particularly telescopic nailing, in these patients. Patients were grouped by treatment type, and outcomes, including fracture frequency, deformity recurrence, need for reintervention, and functional status, were compared. Results showed that telescopic nailing significantly reduced the number of fractures, deformities, and reinterventions compared to traditional rodding techniques. The study concludes that telescopic nails are beneficial in preventing deformities and fractures while allowing normal growth, making them the preferred method for managing patients with OI.

## Introduction

Osteogenesis imperfecta (OI), or "brittle bone disease," is a rare hereditary condition characterized by bone fragility due to defective collagen type I synthesis. It affects approximately one in 15,000 to 20,000 live births, without predilection for sex, ethnicity, or race [1]. Clinical manifestations vary widely, from lethal perinatal forms to milder phenotypes with minimal fractures [2].

Management of OI is complex and requires a lifelong multidisciplinary approach. Advances such as bisphosphonate therapy and telescopic intramedullary nailing have improved the prognosis and quality of life for many patients [3,4]. The current study evaluates the clinical, radiological, and functional outcomes of patients with OI undergoing osteosynthesis, with a focus on telescopic nailing, and identifies the technical considerations and complications associated with this procedure.

## Materials and methods

Study design and population

We conducted a retrospective analysis of 42 pediatric patients diagnosed with OI and treated surgically between March 2009 and December 2022 at the pediatric traumatology and orthopedics department.

Data collection

Demographic and clinical data, including age, sex, autonomy (defined as the ability to walk independently without external support), family history, and initial presentation, were extracted from patient records. Radiological assessments included X-rays, dual-energy X-ray absorptiometry (DXA) scans, and, in some cases, CT imaging. Information regarding medical (bisphosphonate use) and surgical interventions was documented. DXA scans were performed biannually to monitor bone mineral density. Bisphosphonate therapy, initiated postoperatively in most cases, was administered intravenously every three to six months using pamidronate or zoledronic acid.

Surgical techniques

Two surgical modalities were used: traditional intramedullary rodding with solid rods and telescopic nailing using Bailey-Dubow or Fassier-Duval systems. The choice of implant depended on the patient's age, bone size, and the severity of the deformity. Telescopic nails, which accommodate bone growth, were preferred for children over two years of age with suitable bone morphology. Revisions were planned when patients approached the growth limit of the rod or when mechanical failure (e.g., disengagement or sliding failure) occurred.

Follow-up and evaluation

Patients were followed for a mean of 82 months (telescopic group) and 90 months (rodding group). Evaluations were conducted at two weeks, six weeks, three months, six months, and annually, including both clinical and radiological assessments. Functional outcomes were measured using the Pediatric Outcomes Data Collection Instrument. Physiotherapy was initiated during early postoperative weeks, focusing on mobilization, progressive weight-bearing, and muscle strengthening. Nutritional support was provided as needed, with a focus on ensuring adequate calcium and vitamin D intake.

Statistical analysis

Data were analyzed using JAMOVI software (JAMOVI, Sydney, Australia). Continuous variables were expressed as means and standard deviations; categorical variables were reported as frequencies and percentages. Paired t-tests, McNemar's tests, and chi-square tests were used where appropriate, with significance set at p<0.05.

## Results

Demographic and clinical characteristics

**Figure 1:**
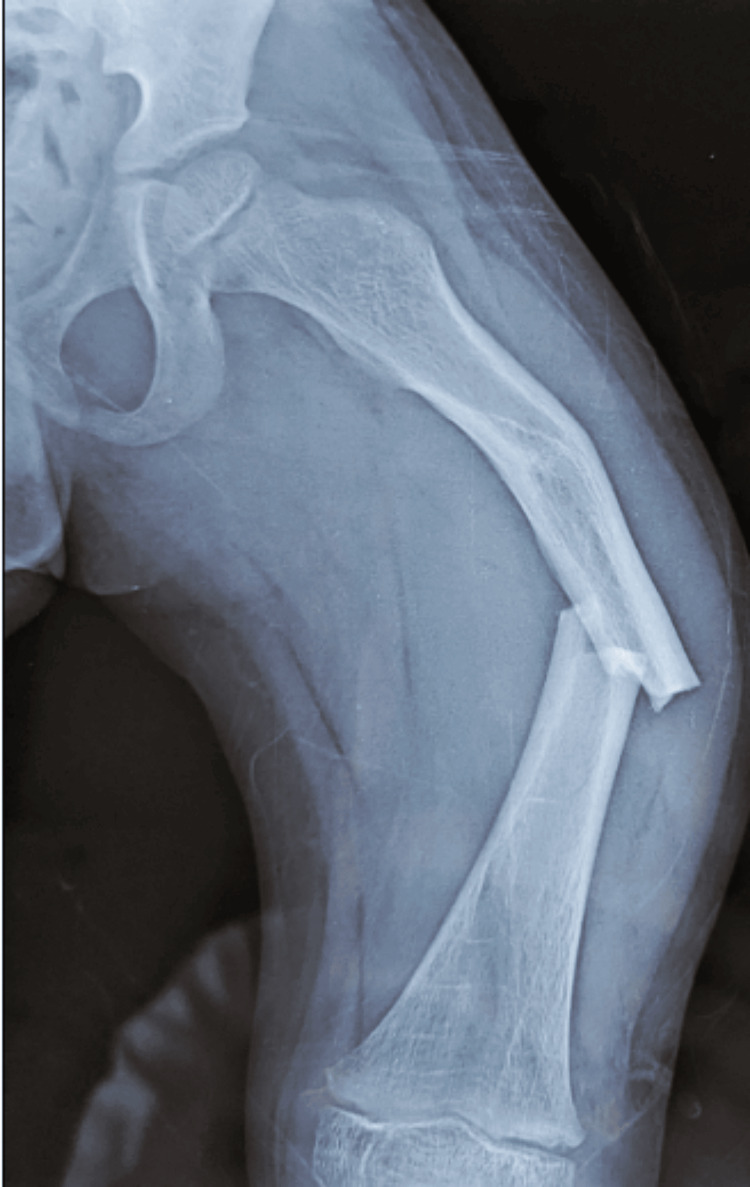
Anterior radiograph of the femur in a child with OI showing a displaced transverse fracture This image highlights the bone fragility characteristic of OI, with the fracture occurring despite minimal trauma. OI: osteogenesis imperfecta

**Figure 2:**
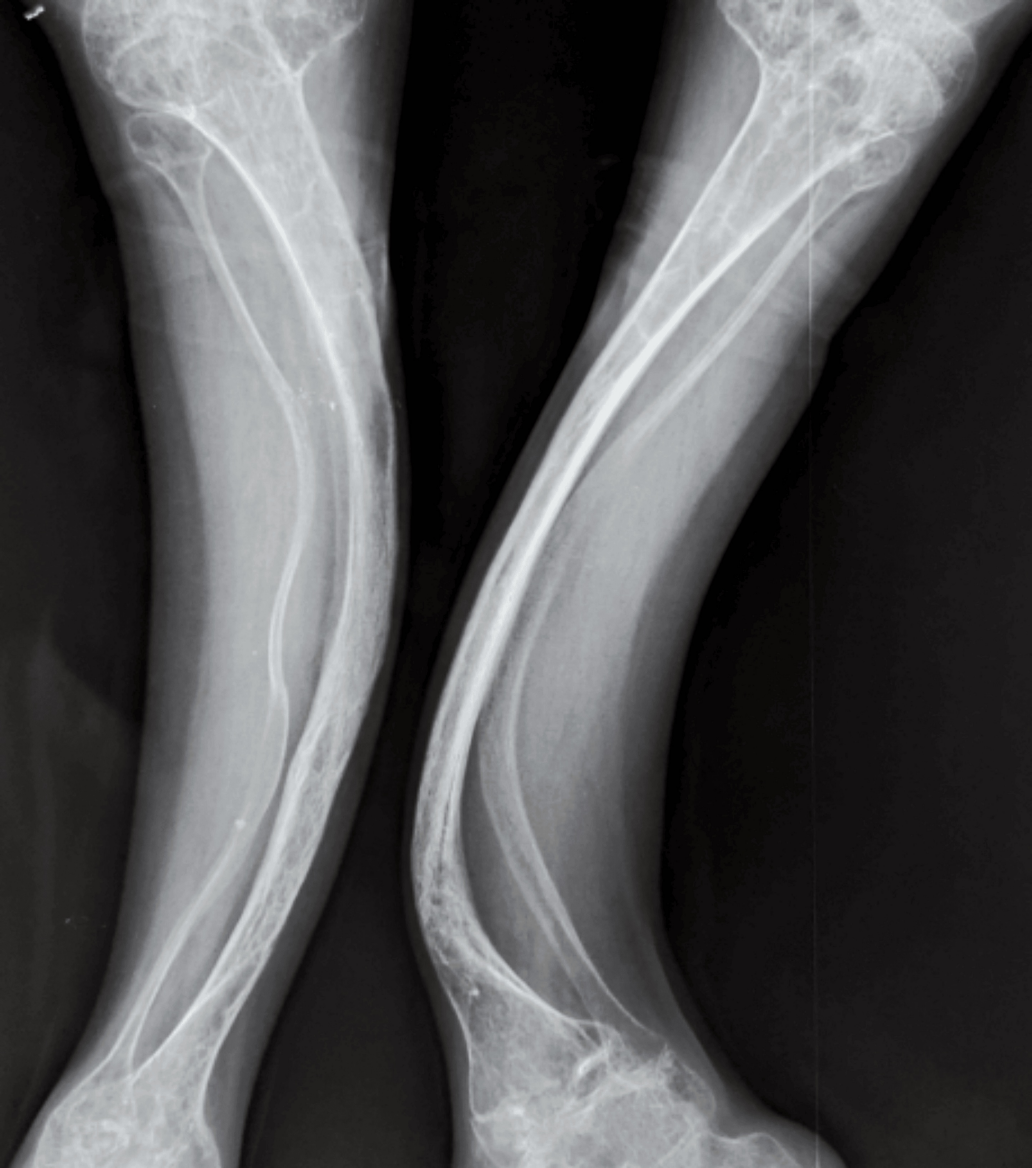
Frontal radiograph of both tibias revealing “saber blade” deformities These bowing deformities are common in children with untreated or advanced OI, reflecting long-standing biomechanical stress on fragile bones. OI: osteogenesis imperfecta

The demographic and clinical characteristics of the 42 patients were as follows: 22 were girls (52%) and 20 were boys (48%), with a mean age of four years. Most cases (76%) were identified after multiple fractures (Figure 1), while 21.5% were diagnosed due to lower limb deformities (Figure 2). The initial presentation most commonly included pain and difficulty ambulating following minor trauma.

**Table 1:** Demographic and clinical characteristics of the study population This table summarizes key baseline features of the 42 patients with OI, including sex distribution, mean age, initial autonomy status (defined as the ability to ambulate independently without assistive devices), and circumstances of diagnosis, such as the presence of multiple fractures or limb deformities. M: male, F: female, OI: osteogenesis imperfecta

Characteristic	Value
Total patients	42
Sex ratio (F:M)	22:20
Mean age (years)	4
Autonomy at presentation (%)	29
Multiple fractures at discovery (%)	76
Lower limb deformities at discovery (%)	21.5

Treatment modalities

**Figure 3:**
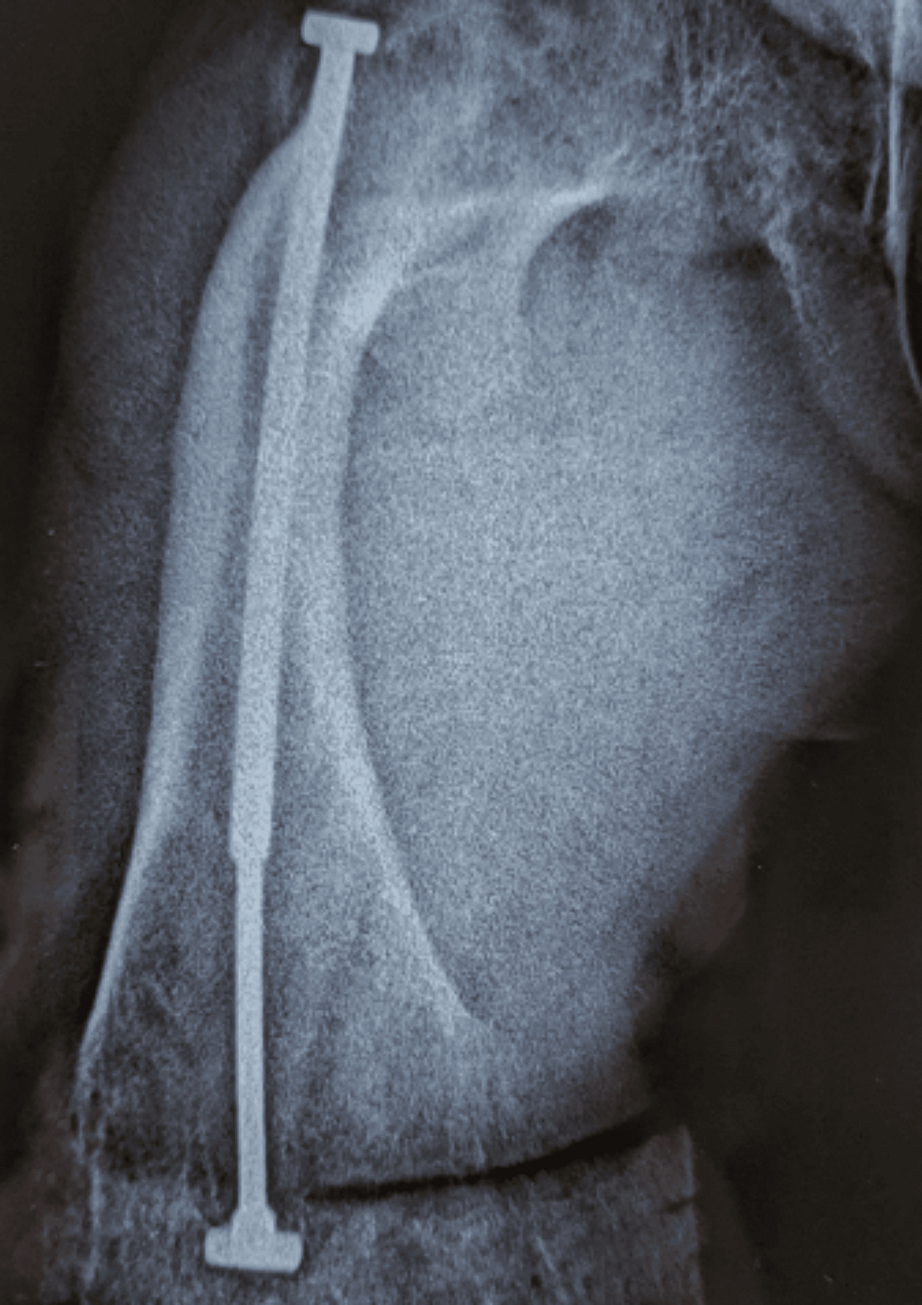
Radiograph demonstrating successful intramedullary rodding with good epiphyseal fixation This fixation allowed the femur to remain protected throughout growth without further deformity or fracture.

All patients underwent surgical treatment: 30 (71%) received telescopic nails, and 12 (29%) underwent intramedullary rodding (Figure 3). Bisphosphonates were administered to 22 patients.

Postoperative outcomes

Table 2 compares postoperative results between groups. The telescopic nailing group demonstrated significantly lower rates of fractures (33.33% vs. 75%), deformities (23.33% vs. 50%), and reinterventions (23.33% vs. 100%).

**Table 2:** Comparison of postoperative outcomes between intramedullary rodding and telescopic nailing. This table contrasts the rates of postoperative fractures, deformities, and surgical reinterventions in the two treatment groups. Telescopic nailing demonstrates superior outcomes across all categories compared to traditional intramedullary rodding.

Outcome	Intramedullary rodding (n=12)	Telescopic mailing (n=30)
Postoperative fractures (%)	75	33.33
Postoperative deformities (%)	50	23.33
Reinterventions (%)	100	23.33

Detailed Group Outcomes

**Figure 4:**
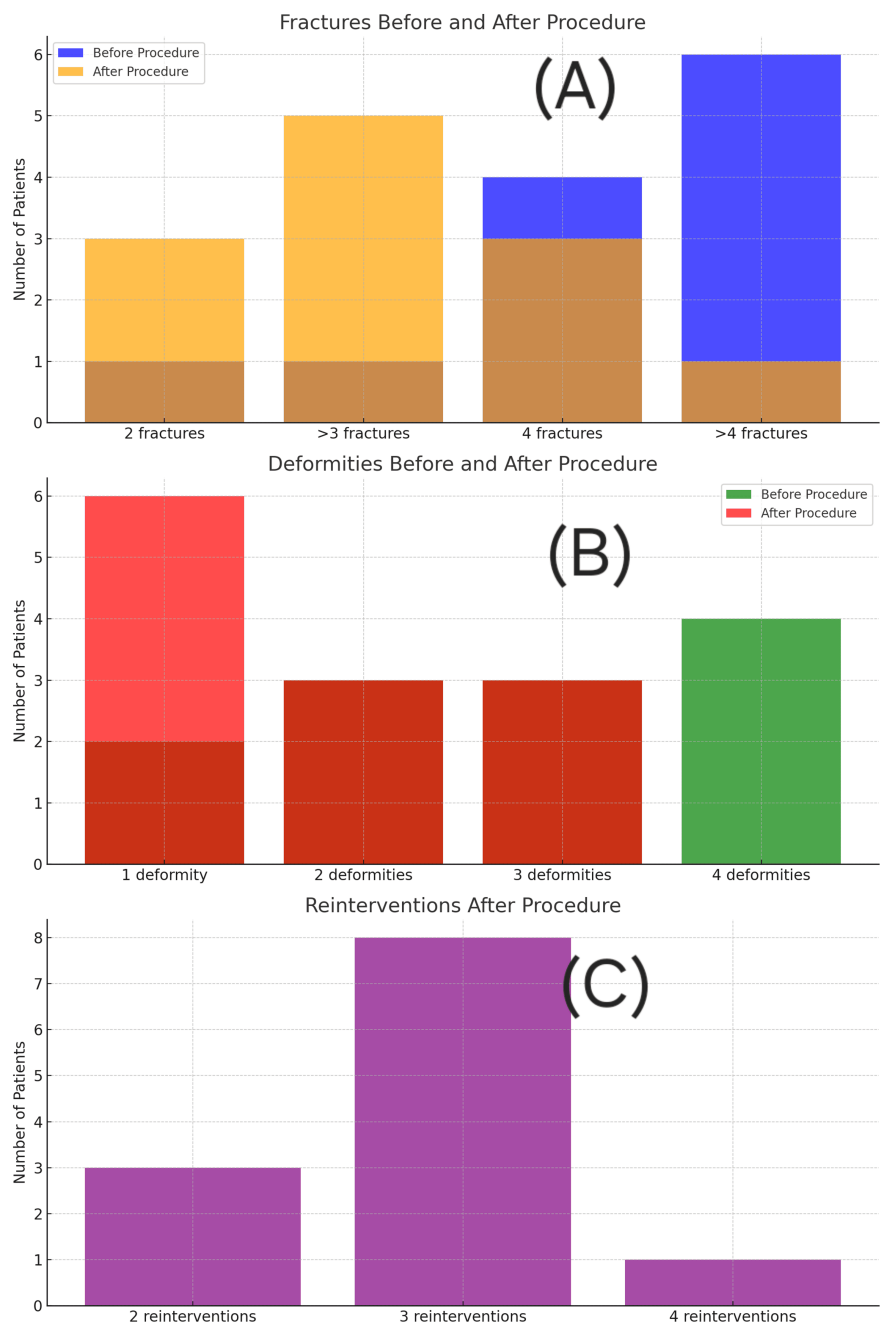
Bar graphs representing fracture count, deformities, and reintervention rates in the intramedullary rodding group. (A) Preoperative vs. postoperative fracture count. (B) Number of deformities before and after surgery. (C) Frequency and number of surgical reinterventions. Each panel illustrates clinical improvement, but with higher complication rates than telescopic nailing.

**Figure 5:**
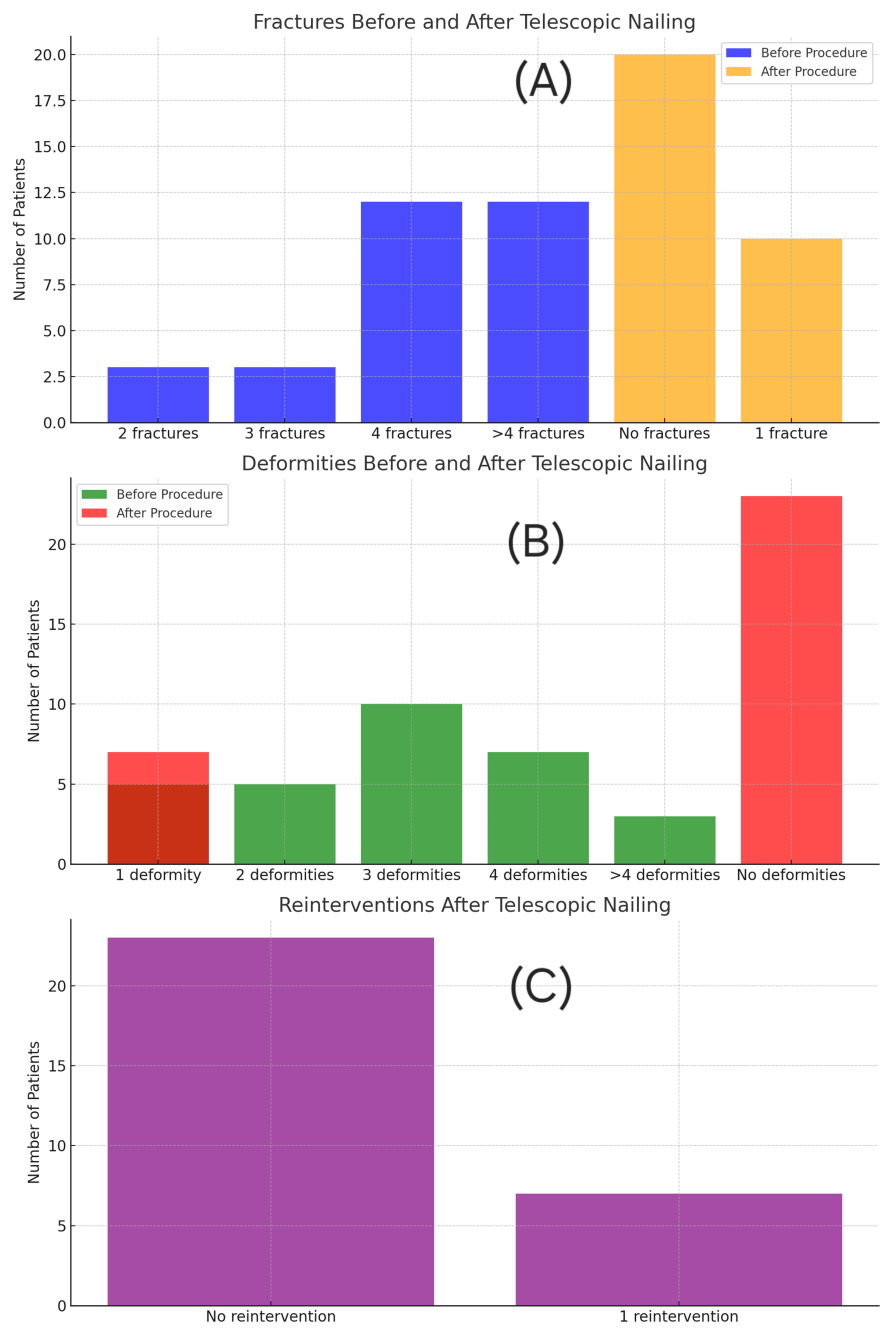
Bar graphs representing outcomes in the telescopic nailing group (A) Fracture incidence before and after telescopic nailing. (B) Deformity count per patient pre- and post-surgery. (C) Number of reinterventions required postoperatively. Note the improved outcomes in all categories compared to Figure 4.

Figures 4-5 illustrate the distribution of fractures, deformities, and reinterventions before and after surgery for each group. The telescopic group had better outcomes across all categories.

Complications

**Figure 6:**
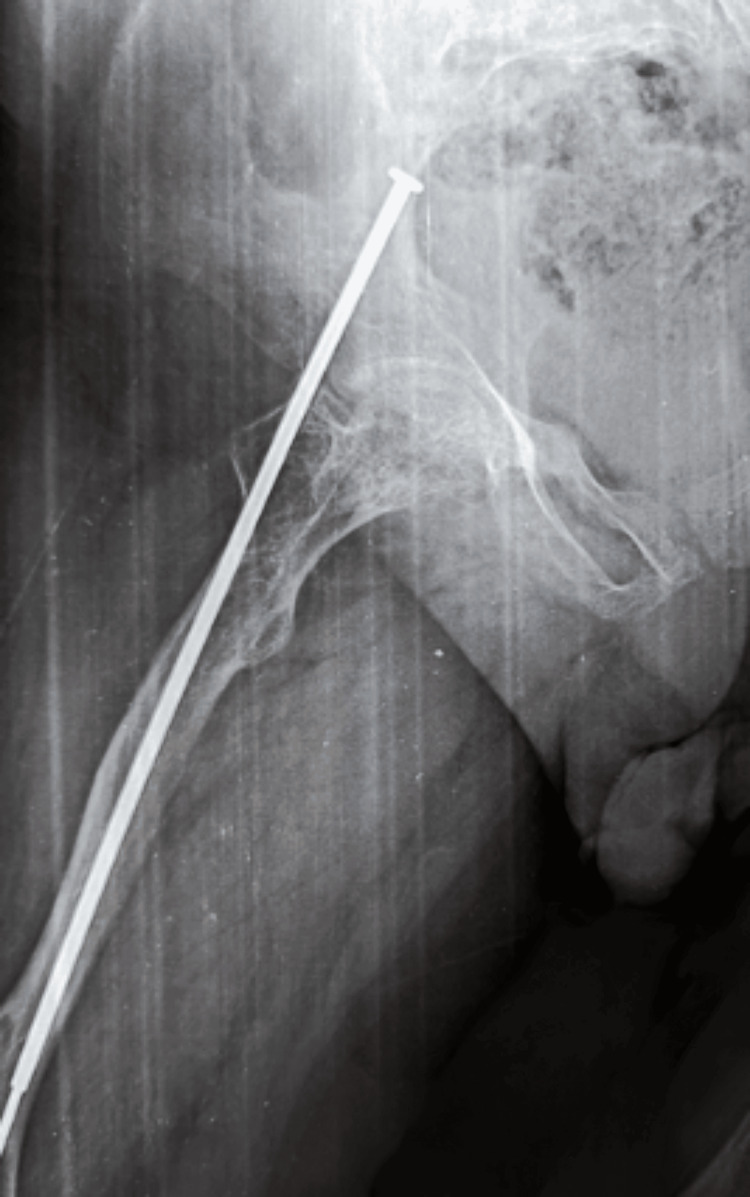
Radiograph showing telescopic nail migration into surrounding soft tissues This complication may occur due to poor initial anchorage or trauma, highlighting the need for meticulous technique.

**Figure 7:**
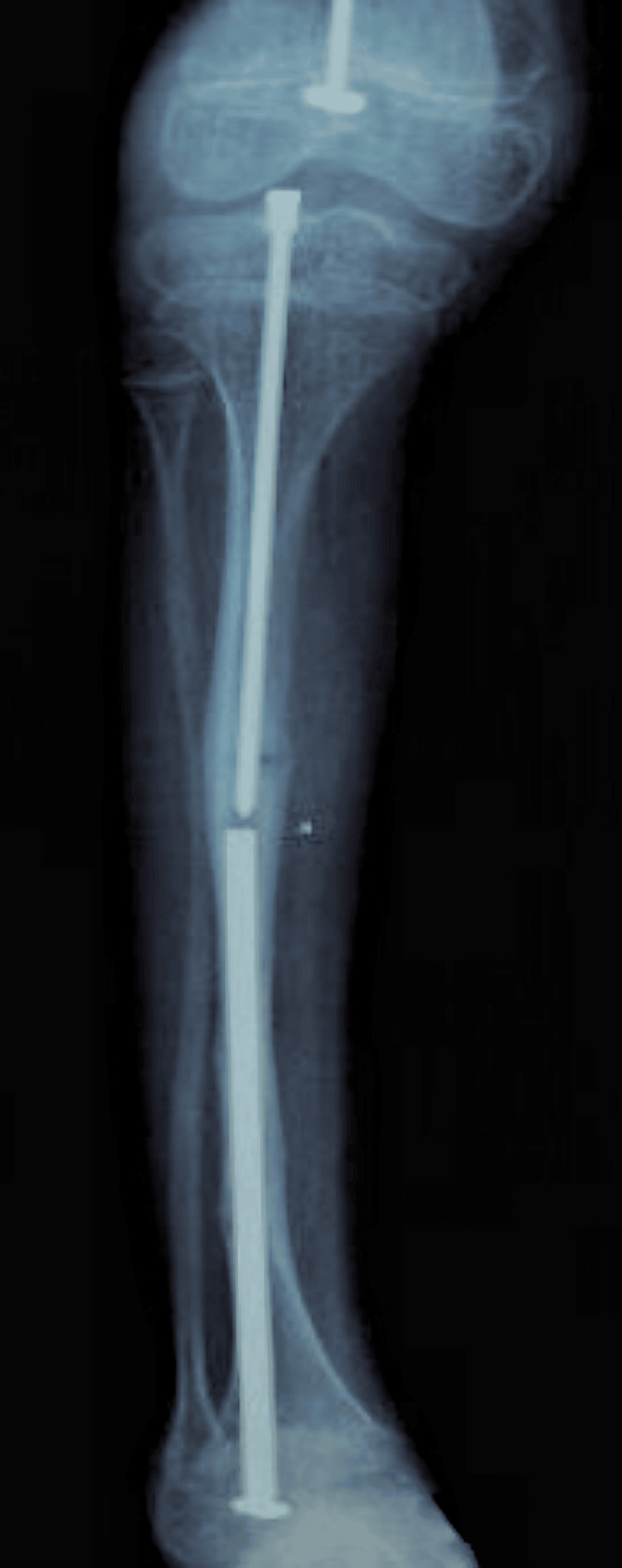
Radiograph showing complete premature opening of the telescopic nail This outcome may compromise stability and require revision surgery.

**Figure 8:**
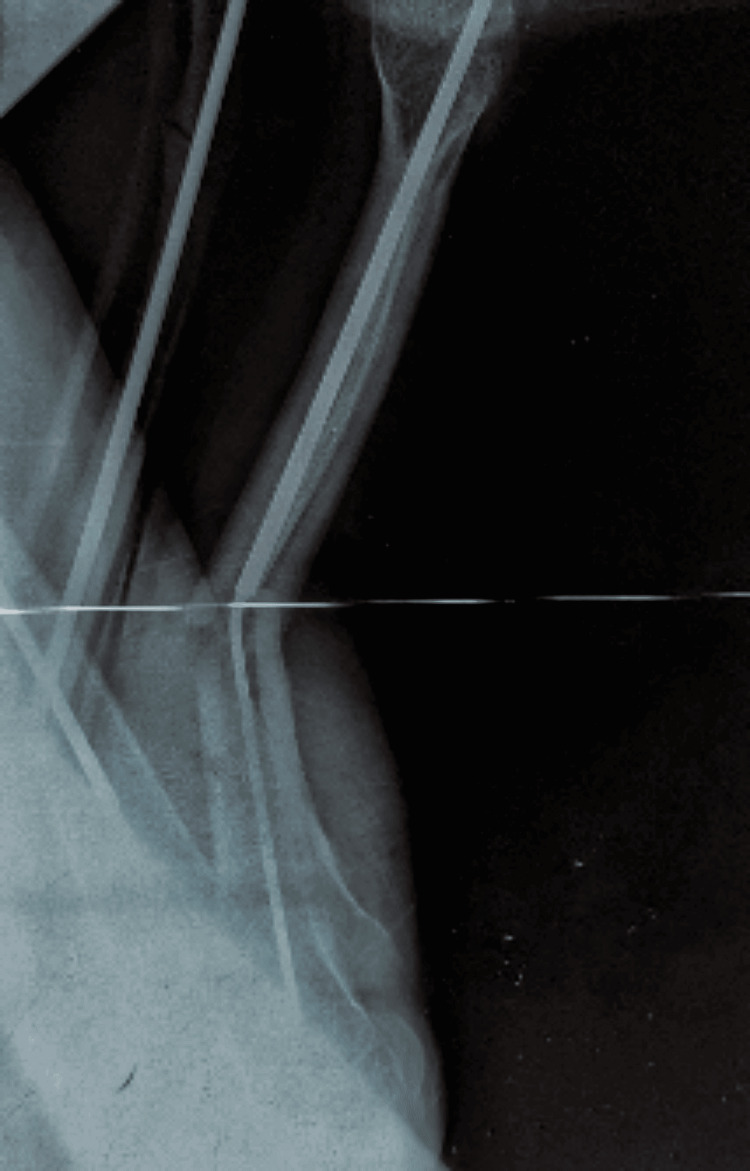
Radiograph showing deformation of the telescopic nail following trauma Illustrates the mechanical limitations of the implant under high stress.

**Figure 9:**
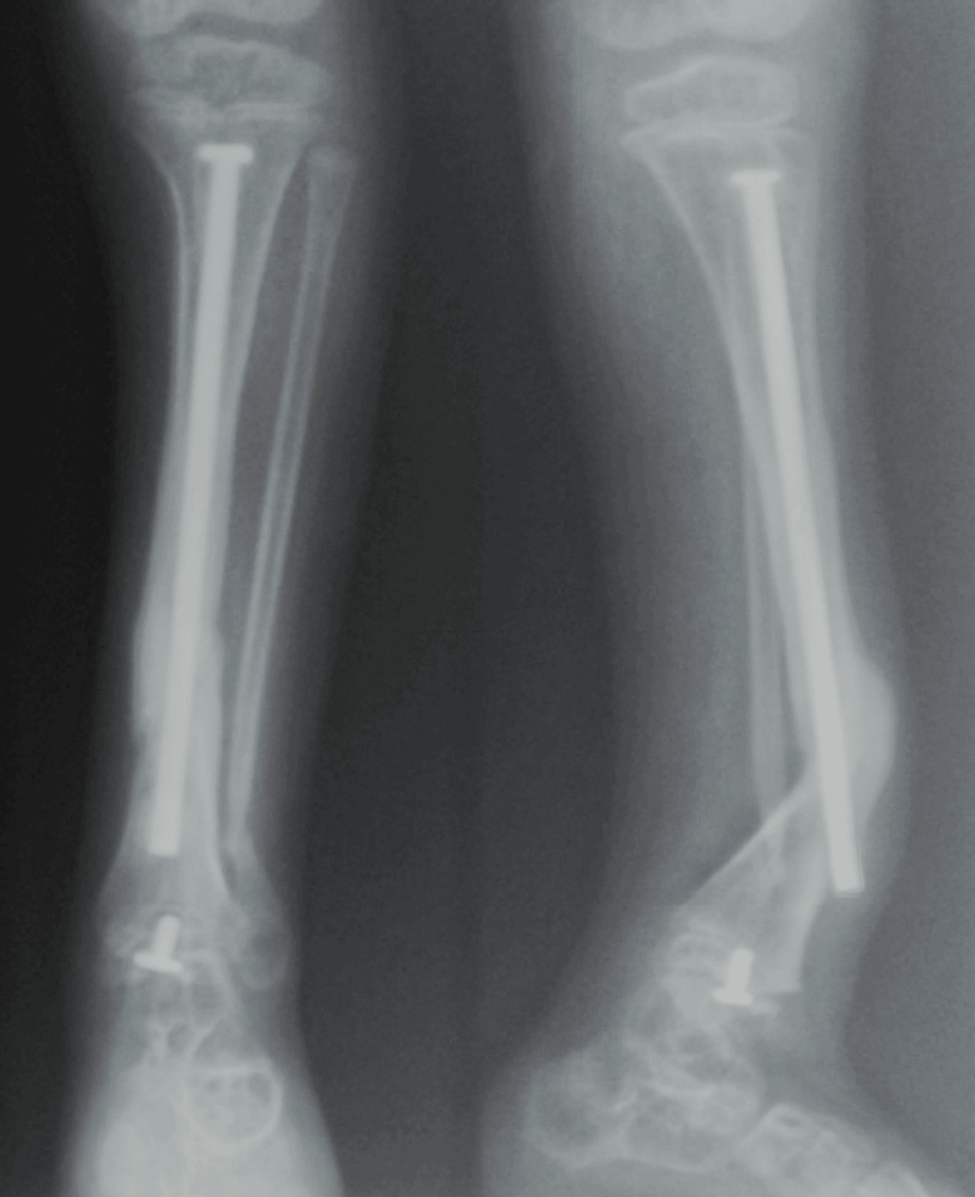
Radiograph showing T-piece migration in a telescopic nail This complication can affect the telescoping mechanism and lead to implant dysfunction.

Complications in the telescopic group included failure of nail opening, migration into soft tissues (Figure 6), complete opening (Figure 7), proximal eccentricity, deformation of the nail following trauma (Figure 8), and T-piece disengagement (Figure 9). In our cohort, nail migration referred to disengagement of the telescopic segments or failure of the sliding mechanism during longitudinal bone growth.

Functional outcomes

Of the 30 previously non-autonomous patients, 21 became independently ambulatory, increasing autonomy to 70%. Seven patients required assistive devices, while two remained non-autonomous. Ambulatory status was determined by the ability to walk independently without external support, verified through outpatient assessment and physiotherapy reports.

## Discussion

The surgical management of OI is evolving, and our data reinforce the superiority of telescopic nailing. Our demographic findings are consistent with other literature [5-7]. Functional outcomes in our study were also superior to those reported by Ruck et al. [8], where only four of 14 patients achieved autonomy. This comparison underscores the importance of integrating advanced surgical interventions early in the treatment process. It is also worth noting that OI affects both sexes equally, and any observed differences in sex distribution are likely due to sampling variation rather than a true predilection [9,10].

Our results align with studies by Spahn et al. and Ruck et al., showing that telescopic nails provide durable support, reduce complications, and promote mobility [7,8]. By enabling bone growth and early weight-bearing, they significantly enhance recovery.

Nonetheless, these implants are not without risks. As described by Fassier [11] and others [12-14], complications such as implant migration and technical failure require vigilance and precise surgical technique. The marked improvement in autonomy (78.57%) surpasses that reported by Lim et al. [14], likely due to the broader use of telescopic nails and bisphosphonates in our cohort.

Medical management with bisphosphonates increases bone density, reduces fracture rates, and enhances quality of life [3]. Combining this therapy with surgery optimizes outcomes. Early intervention is critical. As shown by Ruck et al., early surgery combined with bisphosphonates yields better functional outcomes [8].

We recommend the telescopic nailing method as the first-line surgical option in pediatric OI due to its adaptability to growth and lower complication rate. Limitations include the retrospective design, small cohort, and absence of a control group. Future studies should be prospective, multicenter, and conducted over a long-term period. Rehabilitation, occupational therapy, and psychosocial support also play vital roles [15]. Our study did not evaluate these, but their importance should be emphasized in future research.

## Conclusions

This retrospective study demonstrates the clear benefits of telescopic nailing in reducing postoperative fractures, deformities, and reinterventions in children with OI. Telescopic implants accommodate bone growth and provide lasting support, leading to improved functional outcomes. When combined with bisphosphonate therapy, this approach significantly enhances patient autonomy and quality of life. Future research should focus on prospective, long-term studies to refine best practices and expand multidisciplinary management strategies for patients with OI.
